# A systematic review and meta-analysis of Macroplastique for treating female stress urinary incontinence

**DOI:** 10.1007/s00192-012-1825-9

**Published:** 2012-06-15

**Authors:** Gamal M. Ghoniem, Christopher J. Miller

**Affiliations:** 1 Department of Urology, University of California, Irvine, Orange, CA USA; 2 The Integra Group, Brooklyn Park, MN USA; 3 Division of Female Urology, Pelvic Reconstructive Surgery and Voiding Dysfunction, University of California, Irvine, 333 City Blvd West, Suite 2100, Orange, CA 92868 USA

**Keywords:** Long-term effectiveness, Macroplastique, Meta-analysis, Randomized controlled trial, Silicone injection, Stress urinary incontinence

## Abstract

**Introduction and hypothesis:**

Macroplastique® (polydimethylsiloxane injection) is a minimally invasive urethral bulking agent with global clinical literature describing its use over 20 years. This study critically assessed the safety and effectiveness outcomes for adult women treated with Macroplastique for stress urinary incontinence (SUI) through a systematic review and meta-analysis.

**Methods:**

A systematic review of the scientific literature from 1990 to 2010 was conducted in accordance with the Preferred Reporting Items for Systematic Reviews and Meta-Analyses (PRISMA) statement to quantitatively summarize the safety and effectiveness of Macroplastique for female SUI. A total of 958 patients from 23 cohorts were eligible for inclusion and were analyzed. Random-effects models were used to estimate the improvement and cure rates following treatment at three time periods: short-term (<6 months), mid-term (6–18 months), and long-term (>18 months). Expanded models assessed the effect of reinjection rate on successful treatment outcomes. Adverse event rates were aggregated and reported.

**Results:**

Improvement rates were 75 % [95 % confidence interval (CI), 69–81] in the short-term, 73 % (95 % CI, 62–83) in the mid-term, and 64 % (95 % CI, 57–71) long-term. Cure/dry rates were 43 % (95 % CI, 33–54), 37 % (95 % CI, 28–46), and 36 % (95 % CI, 27–46) over the same respective follow-up periods. Higher study reinjection rates were associated with improved long-term SUI outcomes. No serious adverse events were reported.

**Conclusions:**

This quantitative review supports Macroplastique as an effective, durable, and safe treatment option for female SUI. Meta-analytic evidence suggests that long-term therapeutic benefit is frequently maintained, with some patients requiring reinjection.

## Introduction

Stress urinary incontinence (SUI) is a socially, emotionally, and physically devastating condition affecting millions of women worldwide. SUI is self-reported in up to 25 % of the adult female population in the United States [1]. Standard treatments include pelvic floor muscle exercise regimes, biofeedback, urethral slings, tension-free vaginal tapes (TVT), and urethral bulking agents (UBAs). Midurethral slings are the most frequently performed and effective surgical intervention for SUI. UBAs have been recommended by the American Urology Association for patients who do not wish to undergo a more invasive surgery, elderly patients, and patients at higher risk for anesthetic complications [2]. Guidance from the Society of Obstetricians and Gynaecologists of Canada Urogynaecology Committee, the American Congress of Obstetricians and Gynecologists, and the National Institute for Health and Clinical Excellence (NICE), among others, directs patients with significantly decreased urethral mobility and/or patients who have failed appropriate conservative therapy to consider periurethral bulking agents as one of several treatment options [3–5].

In 2011, glutaraldehyde-treated bovine collagen (Contigen®, CR Bard), the UBA with the longest history of use for female SUI, was withdrawn from worldwide availability, posing a pressing need for the urological community to evaluate alternative UBAs. New synthetic UBAs were developed; however, several—such as polytetrafluoroethylene, dextranomer/hyaluronic acid, and ethylene vinyl alcohol copolymer—were subsequently withdrawn or discontinued for safety or efficacy concerns. Currently, carbon-coated beads, calcium hydroxylapatite (CaHA), and polydimethylsiloxane are marketed to treat female SUI in the United States, whereas autologous cellular therapy, autologous fat, and autologous ear chondrocytes lack sufficient data on efficacy and safety and are considered investigational. Continence outcomes from randomized controlled trials of UBAs suggest that the efficacy of carbon-coated spheres, CaHA, and polydimethylsiloxane is similar to cross-linked collagen [6–8].

**Fig. 1:**
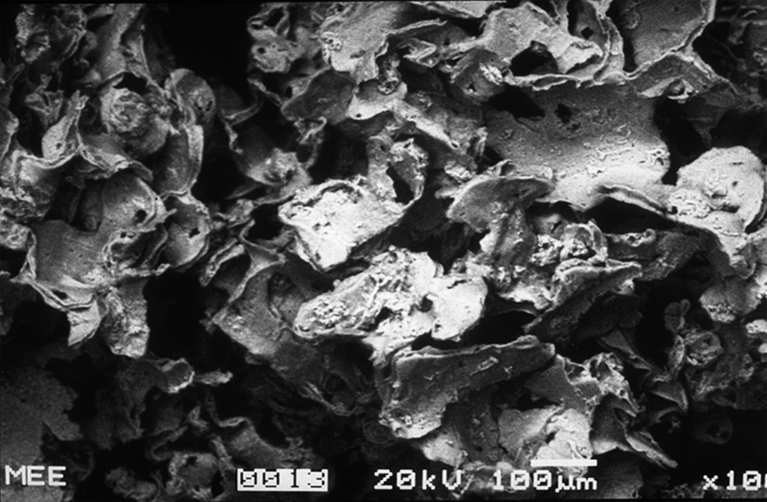
Scanning electron microscopy image of Macroplastique implants

Polydimethylsiloxane (Macroplastique®, Uroplasty, Inc., Minnetonka, MN, USA) has a long history in the treatment of female SUI, primarily in Europe, where it has been used since 1991 and is the leading UBA outside the United States. Macroplastique is indicated to treat SUI primarily due to intrinsic sphincter deficiency (ISD). [Medicare, a federal program in the United States that provides for certain health care expenses for people ≥65, defines ISD in women with SUI as abdominal leak point pressures (ALPP) of ≤100 cm H_2_O without urethral hypermobility.] Macroplastique is nonallergenic, supplied in a ready to use form, and may be carried out in an office setting under local anesthesia. Macroplastique is comprised of highly textured silicone elastomer implants suspended in a polyvinylpyrrolidone (PVP) carrier matrix (Fig. 1). Macroplastique is implanted at several positions around the urethra, under direct vision with a cystoscope, approximately 1 cm distal to the bladder neck to reduce urine leakage from the bladder by bulking and coapting the urethral tissue left open by the weakened sphincter. The PVP carrier matrix is exchanged for tissue fluids containing host fibroblasts. The large, heavily textured elastomer implants are conducive to tissue in-growth, agglomerating and creating a bolus surrounded and infiltrated by host collagen. The fibrous encapsulation of the bolus anchors it within the space between the lamina propria and the urethral muscularis, preventing subsequent implant movement or migration. After implantation, the PVP carrier is resorbed by the reticuloendothelial system and excreted via glomerular filtration without being metabolized.

In 2003, a systematic review of Macroplastique concluded too few prospective, randomized studies had been conducted to reach definitive conclusions about its effectiveness [9]. Since then, a randomized controlled trial (RCT) comparing the effectiveness of Macroplastique to collagen was carried out [8], leading to marketing approval in the United States in 2006. Additionally, three other randomized studies comparing Macroplastique to pelvic floor muscle exercise therapy, pubovaginal sling surgery, and porcine dermal implant injection have been published [10–12]. These trials are complemented by several prospective studies of small cohorts and descriptive reports from clinical practice.

The goal of our study was to systematically review the scientific literature and meta-analyze Macroplastique treatment outcomes for treatment of adult female SUI over time. Additionally, we sought to investigate the effect of reinjection rates on improvement and cure rates and to aggregate reports of adverse events (AEs) to characterize treatment safety.

## Methods

### Study design and data sources

This systematic review and meta-analysis was conducted using the guidelines set forth in the Preferred Reporting Items for Systematic Reviews and Meta-Analyses (PRISMA) statement [13]. We searched Ovid MEDLINE, PubMed, and the Cochrane Library databases from January 1990 to June 2010 and checked the references of systematic reviews for studies reporting Macroplastique treatment in women with SUI. Search terms were all pairwise combinations of the following terms: “Macroplastique,” “stress urinary incontinence,” and “silicone injection,” as well as “Macroplastique” alone.

### Inclusion and exclusion criteria

Two statistical consultants (The Integra Group, Brooklyn Park, MN, USA) evaluated articles independently and in duplicate, and disagreements were resolved by consensus. Candidates for inclusion were peer-reviewed publications of RCTs and prospective, observational, or cohort studies reporting treatment outcomes and/or AEs related Macroplastique treatment for female SUI. Case studies, letter reports, reviews, and animal studies were excluded. To avoid bias, we also excluded statistics for which 25 % of the sample was lost to follow-up. In cases in which data from a particular study sample appeared in more than one publication, the article with the most complete follow-up data was used. We imposed no search restrictions based on language.

### Data extraction and quality assessment

Data were extracted into a database by the second author and were double-checked independently by another statistical consultant for accuracy. The metric for assessing treatment efficacy varied between studies (e.g., Stamey incontinence grade, physician and patient ratings of improvement, pad weight), so we characterized treatment success according to the proportion of the study sample “cured” (i.e., defined as no symptoms of SUI, Stamey grade 0, or dryness) or “improved” (i.e., defined as cured or significant improvement in symptoms from baseline). In cases in which physician and patient assessments were reported, physician evaluations were used.

Follow-up periods from published studies varied from 1 month to >5years. Improvement and cure rates were classified into three time strata: short-term (<6 months), mid-term (6–18 months), and long-term (>18 months). The proportion of patients improved and cured was meta-analyzed for each time stratum. For retrospective studies in which improvement and cure rates were not collected at a prespecified time, we used the sample’s mean follow-up time to classify the time stratum. Where available, data on reinjection rates within studies were extracted to explore potential relationships with treatment success. Reported AEs were also extracted; however, clinical definitions varied too greatly between studies for meta-analysis. We report the median value and interquartile range (IQR) for AE rates across studies.

We evaluated methodological quality of all articles meeting inclusion criteria in several domains:

1. description of the study population and selection method;
2. description of potential confounders (e.g., urethral hypermobility and history of incontinence surgery);
3. reporting of study location(s) and enrollment/treatment dates;
4. description of treatment exposure (e.g., injection method, average volume injected, number of treatments, reinjection rates);
5. appropriate statistical analysis;
6. description of randomization, blinding, and Institutional Review Board approval, where applicable;
7. description of loss to follow-up and the handling of missing data;
8. reporting of AEs.

### Statistical analysis

Significant heterogeneity between study outcomes was anticipated due to the small sample sizes of several studies and different designs. Therefore, we used random-effects (RE) linear regression models to estimate the proportions of the sample improved and cured at each of the three time strata. We applied an arcsine square-root transformation to stabilize variance estimates where necessary to ensure confidence limits were not estimated to be <0 % or >100 % [14]. Results from models using the arcsine-transformed data are reported back-transformed, so interpretation is identical for all models regardless of data transformation. Forest plots show study-specific and model estimates for each endpoint and indicate whether the arcsine transformation was applied. Expanded RE models were used to explore the effect of study reinjection rates on improvement and cure rates. Study heterogeneity, which describes the variation between study outcomes not due to chance variation, was measured using I^2^ (the percent of total variability in study outcomes attributable to heterogeneity) and was tested for significance using Cochran’s* Q* test. Funnel-plot asymmetry, an indicator of potential reporting bias, was assessed with Egger’s test. Statistical analyses were conducted with the metafor package [15] for R, version 2.14.1 (R Development Core Team, 2011).

## Results

### Study and sample characteristics

Sixty-five unique candidate articles were retrieved from all searches, and 23 patient cohorts from 24 published articles [8, 10–12, 16–35] met inclusion criteria and were included in the meta-analysis, for a total of 958 patients. Four patient cohorts were from randomized studies [8, 10–12], nine were prospective observational studies [16–23, 32], and ten were retrospective studies of clinical practice [24–30, 33–35]. We did not include 27 review papers, four case reports, nine studies that did not use Macroplastique, and one animal study. We excluded long-term data from three papers with insufficient follow-up [11, 31, 34]. All studies used a transurethral injection technique with either endoscopic injection or the Macroplastique Implantation System (MIS; Uroplasty BV, Geleen, The Netherlands), which is a nonendoscopic needle guide. Most studies reviewed defined ISD as maximum urethral pressure (MUP) or maximum closing urethral pressure (MUCP) ≤20 cm H_2_O and/or Valsalva’s leak-point pressure (VLPP/ALPP) <60 cm H_2_O.

Summaries of each study, including reported AEs and assessments for risk of bias according to the previously defined criteria, are presented in Table 1. Median age of study samples was 59 years (IQR, 54–65). The median proportion of the study samples that had undergone at least one previous surgery for urinary incontinence was 42 % (IQR, 30–70).

**Table 1:** Summary of studies included in the meta-analysis

Authors	Study design	No. participants	Adverse event rates	Risk of bias assessment
Ghoniem et al. [8]	Randomized study, blinded	122	24 % urinary tract infection, 12 % frequency, 9 % dysuria, 7 % retention, 5 % voiding	No evidence of bias
ter Meulen et al. [10]	Randomized prospective study, unblinded	24	79 % retention, 50 % dysuria, 8 % implant leakage	No evidence of bias
Maher et al. [11]	Randomized prospective study, unblinded	23	9 % urinary tract infections, 4 % voiding	Follow-up at long-term <75 %
Bano et al. [12]	Randomized prospective study, unblinded	24	13 % retention, 1 non-device-related death	No description of sites and dates, sample characteristics
Zullo et al. [16]	Prospective observational study	27	No major intra- or postoperative AEs observed	No description of sample characteristics or reporting of common AEs
Plotti et al. [17]	Prospective observational study	24	No major intra- or postoperative AEs observed	No description of sample characteristics or reporting of common AEs
Zullo et al. [18]	Prospective observational study	61	7 % urgency, 3 % urinary tract infection, 2 % dysuria	No evidence of bias
Tamanini et al. [19], 2006	Prospective observational study	21	100 % dysuria, 14 % retention	No evidence of bias
Radley et al. [20]	Prospective observational study	56	12 % urinary retention, 6 % urinary tract infection	No evidence of bias
Henalla et al. [21]	Prospective observational study	40	18 % retention, 63 % dysuria	No evidence of bias reporting of sample’s mean age.
Barranger et al. [22]	Prospective observational study	21	No major intra- or postoperative AEs observed	No reporting of common AEs
Koelbl et al. [23]	Prospective observational study	32	6 % urinary tract infection	No description of sample characteristics or reporting of common AEs, definition of “cure” not defined
de Tayrac et al. [32]	Prospective study	20	11 % pain, 16 % dysuria, 32 % retention	No evidence of bias
Sander et al. [35]	Retrospective study	53	15 % retention, 13 % urge incontinence, 11 % dysuria	No evidence of bias
Franceschetti et al. [33]	Retrospective study	44	Small loss of blood in one patient due to anesthesia	Reporting of AEs inadequate
Hidar et al. [34]	Retrospective study	25	8 % retention, 16 % implant leakage	Long-term follow-up <75 %
Mourad [24]	Retrospective case series study	48	13 % retention	No reporting of several common AEs; reporting of AEs inadequate
Peeker et al. [25]	Retrospective case series study	20	Most patients reported mild dysuria	Reporting of AEs inadequate
Gürdal et al. [26]	Retrospective review study	29	45 % hematuria, 79 % dysuria, 72 % frequency, 3 % retention	No evidence of bias
Soliman and Evans [27]	Retrospective review study	68	6 % retention	No evidence of bias
Usman and Henalla [28]	Retrospective review study	102	7 % retention, 1 % urinary tract infection	No evidence of bias
Sheriff et al. [29]	Retrospective review study	34	12 % retention, 53 % dysuria, 68 % hematuria, 76 % frequency	No evidence of bias
Harris et al. [30]	Retrospective case series study	40	Almost all patients had dysuria	Reporting of AEs inadequate

Most deviations from the expectation of quality were minor; however, two studies did not provide an adequate definition of improvement and cure. Both were included in meta-analyzed proportions despite contributing outlying data points [23, 25].

### Meta-analysis of success and cure rates

**Fig. 2:**
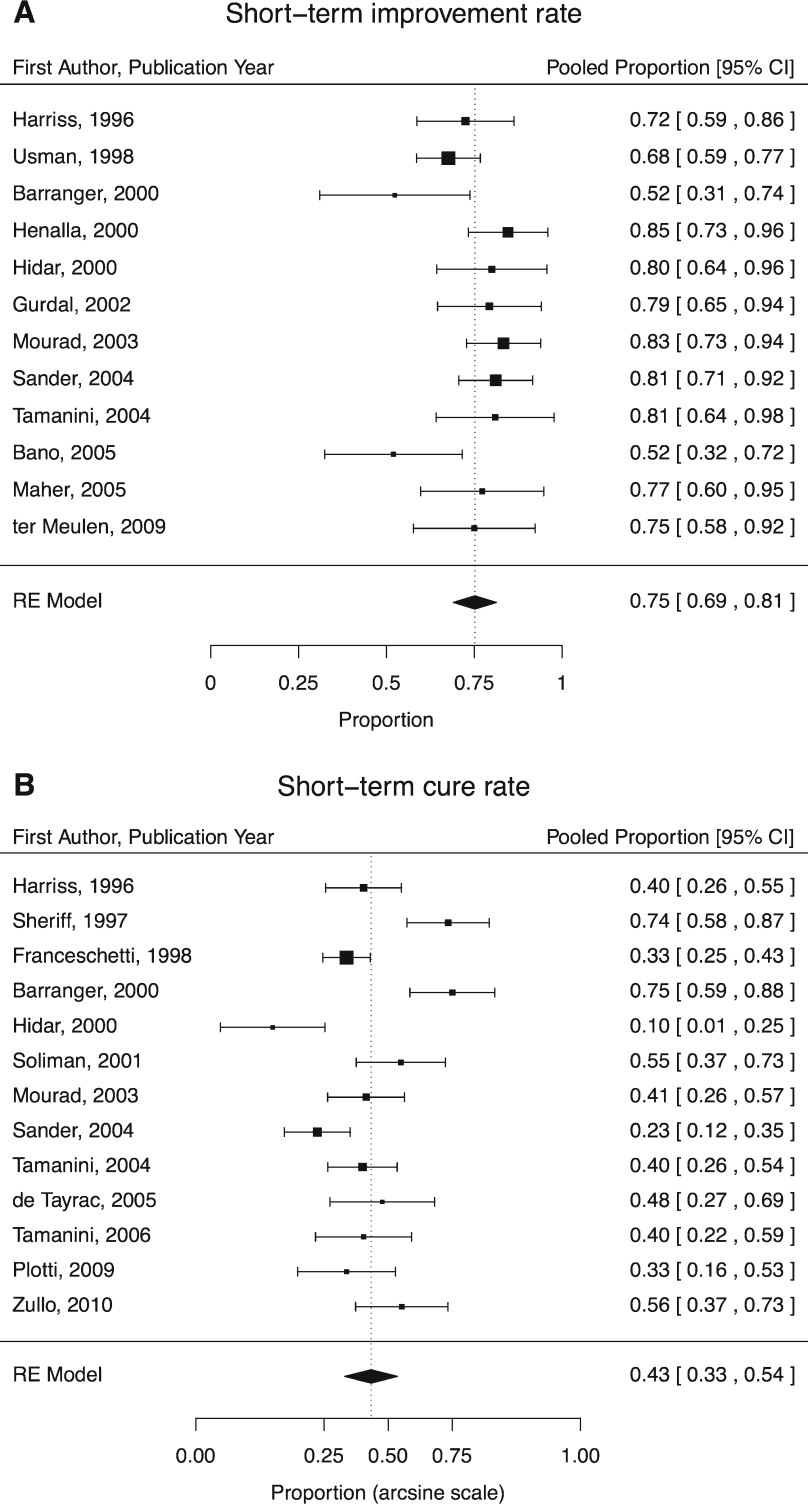
**a, b** Short-term (<6 months) improvement rates (**a**) and cure rates (**b**) sorted by year of publication

Data from 12 studies contributed to the aggregate short-term improvement rate of 75 % [95 % confidence interval (CI), 69–81; (Fig. 2a)]. Thirteen studies contributed to the short-term cure rate of 43 % (95 % CI, 33–54; Fig. 2b). Significant heterogeneity was observed in both short-term improvement (I^2^ = 67 %, *P* = 0.05) and cure (I^2^ = 83 %, *P* < 0.001) rates but not publication bias (both *P* > 0.10).

**Fig. 3:**
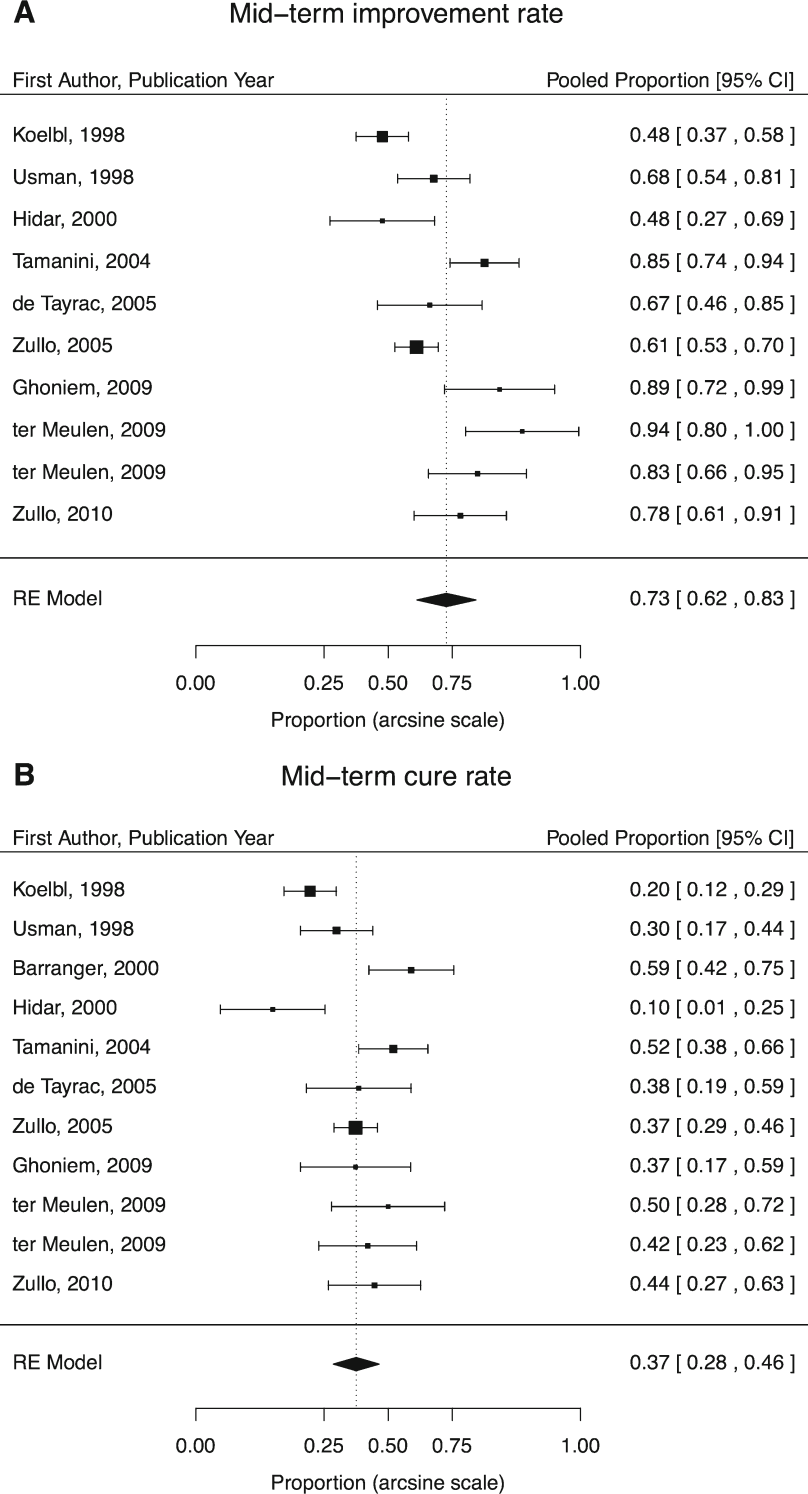
**a, b** Mid-term (6–18 months) improvement rates (**a**) and cure rates (**b**) sorted by year of publication

Data from ten studies contributed to the mid-term improvement rate of 73 % (95 % CI, 62–83; Fig. 3a) and 11 to the cure rate of 37 % (95 % CI, 28–46; Fig. 3b). Significant heterogeneity was detected in mid-term improvement (I^2^ = 83 %, *P* < 0.001) and cure (I^2^ = 72 %, *P* < 0.001) rates but not publication bias (both *P* > 0.10).

**Fig. 4:**
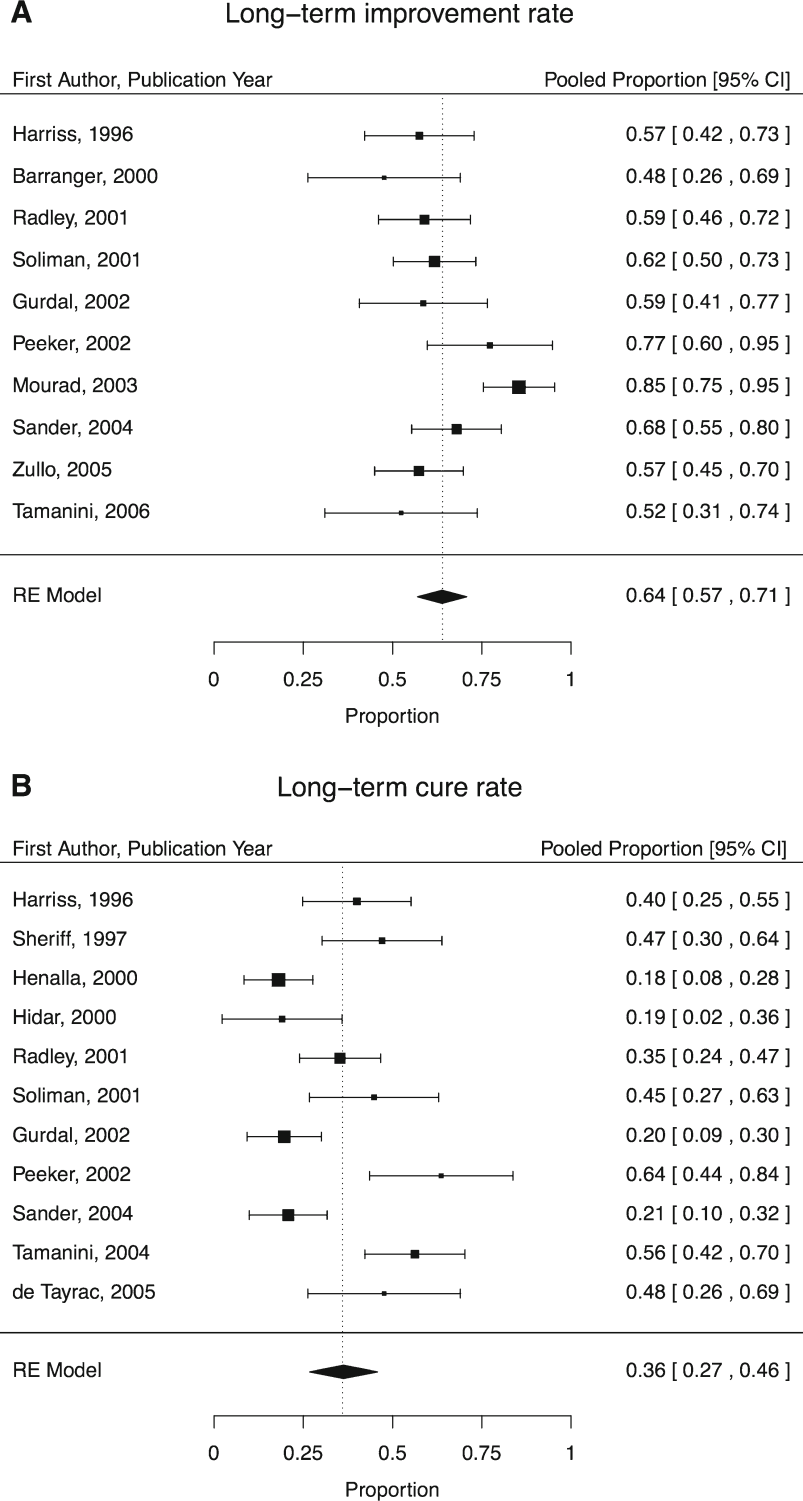
**a, b** Long-term (>18 months) improvement rates (**a**) and cure rates (**b**) sorted by year of publication

Data from ten studies contributed to the aggregate long-term improvement rate of 64 % (95 % CI, 57–71; Fig. 4a) and 11 to the long-term cure rate of 36 % (95 % CI, 27–46; Fig. 4b). Significant heterogeneity was observed for long-term improvement (I^2^ = 56 %, *P* < 0.001) and cure (I^2^ = 79 %, *P* < 0.001) rates. Significant funnel plot asymmetry was detected in the long-term improvement rate, where studies with larger sampling errors (i.e., smaller sample sizes) reported lower improvement rates (*P* = 0.04). Conversely, there was a significant trend of studies with larger sampling errors reporting higher cure rates (*P* = 0.003).

### Effect of reinjection on improvement and cure rates

Seventeen studies reported reinjection rates for patients who had not been cured after the first injection and thus offered reinjections to improve the outcome. Across these 17 studies, the median reinjection rate was 30 % (IQR 14–37). Expanded RE models found that higher reinjection rates were associated with higher improvement rates in long-term but not short-term or mid-term follow-up (both *P* > 0.10). On average, in long-term follow-up, 63 % (95 % CI, 56–70) of patients in a cohort with the typical reinjection rate (30 %) had SUI symptom improvement; each 10 % increase in the proportion of the sample undergoing repeat injections was associated with an additional 4 % (95 % CI, 1–7) of the sample having improved in the long term. Study reinjection rates were not significantly associated with study cure rates at any time point (all *P* > 0.3).

### Adverse events

The median rates for AEs were 7 % (IQR, 5–15) for temporary urinary retention, 7 % (IQR, 4–27) for urge incontinence, 3 % (IQR, 0–8) for urinary tract infections, 50 % (IQR, 11–79) for temporary dysuria, and 45 % (IQR, 8–64) for transient hematuria. There were no reports of extrusion, migration, immune reaction, embolic phenomena, vascular occlusion, or other serious AEs.

## Discussion

This systematic review and meta-analysis of 20 years of global experience in clinical research and practice indicates that Macroplastique is a safe and effective urethral bulking agent for treating women with SUI primarily due to ISD. Pooled results suggest successful outcomes in the short term for 75 % and cures for 43 % of patients. Furthermore, our results suggest that those with initial success frequently sustained therapeutic benefit past 18 months. On average, at the sample level, 85 % of patients sustained their improvement and 84 % sustained their cure, attesting to the durability of Macroplastique therapy. Ghoniem and colleagues followed for 2 years initially successful 12-month patients from the 122 patients treated in the US Investigation Device Exemption (IDE) RCT [31]. Though an insufficient number of patients were followed to include in the our meta-analysis, subcohort results were consistent with the meta-analyzed results reporting that of initially successful patients presenting at 2 years, 56 of 67 patients (84 %) maintained improvement at 2 years and 45 of 67 (67 %) maintained cure.

The long-term reinjection rate was positively associated with treatment outcome on the study level, supporting the clinical practice of reinjection to both sustain improvements and build upon them when the patient is not cured after the first injection. For example, Henalla et al. reported on 14 patients who initially failed treatment; eight (57 %) were cured or significantly improved by a second injection [21]. In addition, four patients who were significantly improved chose to undergo a second injection; three were cured and one remained significantly improved. Our findings considered with clinical evidence suggest that Macroplastique therapy may be approached as a process of treatment and re-evaluation, with the option of repeat injections if initial results are unsatisfactory and with regard to important contraindications such as a fragile urethral mucosal lining.

Though their data were too few for quantitative analysis, a small number of studies reported on patients with SUI of mixed etiology. In the randomized trial by ter Meulen and colleagues, 88 % of patients with SUI and hypermobility (*n* = 24) were cured or markedly improved at 12 months [10]. Findings, however, are mixed, as two studies found no effect of hypermobility on treatment efficacy [11, 19] whereas others found patients with ISD and hypermobility failed at higher rates than those with ISD alone [16, 17]. More research is required to determine the effectiveness of Macroplastique for SUI of mixed etiology.

Several randomized studies compared the relative efficacy of different UBAs primarily with collagen as the comparator. An RCT comparing Macroplastique with collagen (*n* = 247) observed improvement rates of 62 % and 48 % (*P* < 0.001) and cure rates of 37 % and 25 % (*P* < 0.05), respectively, after 12 months [8]. The improvement rate at 12 months for pyrolytic carbon-coated beads (80 %) was not inferior to that of collagen (69 %) in a double-blind trial (*P* = 0.16;* n* = 355) [7]. CaHA was not inferior to collagen in a single-blind trial (*n* = 296), with improvement rates of 63 % and 57 % at 12 months, respectively (*P* = 0.34) [6]. A smaller trial of CaHA and collagen (*n* = 46) reported 80 % improvement in the CaHA group and 62 % in the collagen group after a mean of 32 months of follow-up [36]. A single-blind RCT of dextranomer/hyaluronic acid versus collagen (*n* = 344) documented 12-month improvement rates of 51.2 % and 54.5 %, respectively [37]. An RCT of a copolymer of ethylene vinyl alcohol dissolved in dimethyl sulfoxide compared with collagen (*n* = 249) showed superior 12-month improvement rates over collagen (76 % v. 55 %; *P* < 0.001) [38]. A small RCT of Macroplastique and porcine dermal collagen (*n* = 48) reported improvement in 58 % in the porcine collagen group and 42 % in the Macroplastique group [12].

Macroplastique has a strong safety profile over 20 years, with no record of serious AEs. Importantly, there were no reports of extrusion, migration, or immune reaction in any of the papers identified in our systematic review. The most commonly identified side effects of treatment, transient dysuria and hematuria, are mild. Less frequent AEs included temporary urinary retention, temporary urge incontinence, and urinary tract infection. The RCT comparing Macroplastique with collagen provided the most comprehensive list of AEs among the reviewed publications: urinary tract infection (24 %), dysuria and urgency (9 %), frequency (8 %), and retention (7 %) [8]. These side effects are mild, transient, and easily managed without long-term sequelae for the patient compared with more invasive surgical device procedures, which require long recovery times and an increased risk of serious complications, such as severe pain, organ perforations, hemorrhage, urethral erosion, and vaginal extrusion [39].

Macroplastique is typically administered in an office-based setting with a local anesthetic, providing a simple and low-risk treatment option for women who are not candidates for general anesthesia, women who desire to get pregnant, and patients who desire a short recovery period [2]. Our analysis suggests Macroplastique is most appropriate for women who expect a long-term significant improvement in their SUI symptoms but would be satisfied if not completely cured. A recent study of women’s expectations from SUI interventions suggests most women would consider their treatment successful if they have significant improvement in symptoms (57 %) [40]. In the study of women’s expectations, 71 % of women preferred a minor procedure, such as a UBA, TVT, or transobturator tape (TOT); older women were significantly more likely to choose an office-based procedure. Other women who may prefer Macroplastique are those desiring to have more children or who would prefer a short recovery time [41].

The primary limitation of this meta-analysis is the variability in design among the included studies. Proportions and confidence intervals for some studies were observed both above and below the meta-analyzed result, which led to significant heterogeneity. Many studies had modest samples sizes, which may explain between-study variability in improvement and success rates. This may also suggest that other factors not accounted for in our analysis, such as unmeasured characteristics of the patient population, clinical definitions of cure and improvement, or clinician experience, may mitigate success rates in these studies. Another limitation is that the long-term efficacy rates had significant evidence of potential publication bias. Caution should be exercised in their interpretation, as the long-term success rate tended to be higher in larger studies than in smaller studies, whereas the long-term cure rate tended to be lower in larger studies. Finally, analyses of reinjection efficacy were based on a subset of all studies in the meta-analysis and may not have been adequately powered to detect these associations.

Four randomized trials were found, but none of the meta-analyzed statistics were able to synthesize results from all four studies. Even so, the aggregate statistics agree with and support the results found in the RCTs, with good short-term (75 %) and long-term (64 %) efficacy and safety profiles. Although the studies identified in the systematic review varied in location, design, and setting, their combined results describe a 20-year global history of safe and effective treatment with Macroplastique for adult women with SUI.

## Conclusions

Results of this quantitative review support Macroplastique as an effective and durable treatment option for female SUI. Meta-analysis of all relevant peer-reviewed literature shows that Macroplastique largely sustains its therapeutic benefit in the mid-term and long-term, with some patients requiring reinjection to attain maximal effects. Macroplastique may be best suited to women who desire long-term significant improvement or cure of their SUI and who prefer a minimally invasive procedure with low morbidity and short recovery time.
